# Bi-Assisted CdTe/CdS Hierarchical Nanostructure Growth for Photoconductive Applications 

**DOI:** 10.1186/s11671-015-1037-6

**Published:** 2015-08-19

**Authors:** Kwang Heo, Hyungwoo Lee, Jikang Jian, Dong-Jin Lee, Yongju Park, Changhee Lee, Byung Yang Lee, Seunghun Hong

**Affiliations:** Department of Physics and Astronomy, Seoul National University, Seoul, 151-747 Republic of Korea; Department of Nanotechnology and Advanced Materials Engineering, Sejong University, Seoul, 143-747 Republic of Korea; School of Physics and Optoelectronic Engineering, Guangdong University of Technology, Guangzhou, 510006 China; School of Mechanical Engineering, Korea University, Seoul, 136-713 Republic of Korea; School of Electrical and Computer Engineering, Seoul National University, Seoul, 151-744 Republic of Korea; Department of Biophysics and Chemical Biology, Seoul National University, Seoul, 151-742 Republic of Korea

**Keywords:** Nanowires, Hierarchical structures, Photodetector, Chemical vapor deposition

## Abstract

We developed a method to control the structure of CdTe nanowires by adopting Bi-mixed CdTe powder source to a catalyst-assisted chemical vapor deposition, which allowed us to fabricate CdTe/CdS hierarchical nanostructures. We demonstrated that diverse nanostructures can be grown depending on the combination of the Bi powder and film catalysts. As a proof of concepts, we grew CdTe/CdS branched nanowires for the fabrication of photodetectors. The hierarchical nanostructure-based photodetectors showed an improved photoresponsivity compared to the single CdTe nanowire (NW)-based photodetector. Our strategy can be a simple but powerful method for the development of advanced optoelectronic devices and other practical applications.

**Electronic supplementary material:**

The online version of this article (doi:10.1186/s11671-015-1037-6) contains supplementary material, which is available to authorized users.

## Background

Recently, low-dimensional nanostructures of cadmium chalcogenide (CdS, CdSe, or CdTe) have attracted much attention because of their remarkable electronic and photonic properties [1–7]. In particular, nanowires (NWs) based on cadmium chalcogenide have been considered as versatile building blocks for high-performance optoelectronic devices such as optical waveguides [8], electrical injection lasers [9], photodetectors [10–16], and solar cells [17, 18]. In such optoelectronic devices, a control over the size and the structure of NWs enables the tuning of their energy band gaps and optical properties [19]. Therefore, a lot of studies have been devoted to controlling the structural parameters and morphology of NWs [19–22]. Especially, many researchers fabricated one-dimensional II-VI heterojunctions to develop high-performance photodetectors [23–26]. However, the precise control of the NWs with complex hierarchical nanostructures is still a challenge.

Herein, we report a simple, but versatile, method to control the structure of CdTe NWs by adopting Bi-mixed CdTe powder source to a catalyst-assisted chemical vapor deposition (CVD), resulting in NW-based hierarchical nanostructures with largely enhanced photoconductivity. Using our method, we could build well-defined hierarchical nanostructures such as branched NWs or NWs directly grown on cadmium chalcogenide thin films. As a proof of concepts, we fabricated a photodetector based on CdTe/CdS hierarchical nanostructures which showed much larger photoconductivity than photodetectors based only on CdTe NWs. This result shows that our strategy is a powerful way to develop advanced optoelectronic devices with diverse practical applications.

## Methods

### Bi-Assisted CdTe NW Growth

The NWs were grown in a horizontal tube furnace via a vapor-liquid-solid (VLS) process. CdTe powder (Sigma-Aldrich, purity 99.99 %, size < 250 μm) was mixed with Bi powder (Sigma-Aldrich, purity 99.999 %, ~100 mesh) in a 10:1 weight ratio. The mixed powder was placed at the center of an alumina tube furnace as a source material, while an 8-nm-thick Au or Bi film-coated SiO_2_ substrate was placed at the downstream position of the source material. For the growth of the CdTe NWs, the furnace tube was rapidly heated up to a target temperature of 460~540 °C at a rate of 32.5 °C/min under a constant Ar flow. When the temperature reached the target temperature, the furnace tube was evacuated to a base pressure of 8 × 10^−3^ Torr. Then, the furnace tube was kept at a constant temperature and pressure condition for a controlled time (90~120 min). At the end of the growth process, the tube was thoroughly purged with a constant Ar flow and cooled down slowly.

### CdTe/CdS Hierarchical Nanostructure Growth

Firstly, single-crystalline CdS NWs were grown on a catalyst-coated SiO_2_ substrate using a VLS method. The CdS powder source was placed at the center of the alumina tube, and the substrates with Au catalyst film were placed at the downstream position of the source material. After evacuating the tube (base pressure of 10^−6^ Torr) and purging it with high-purity argon (Ar) for 90 min, the furnace was rapidly heated to 650 °C under a constant Ar flow with its flow rate of 300 sccm. When the temperature reached 650 °C, the tube was evacuated to the base pressure, and then, the tube was kept at this temperature for 40 min. After 40 min, the tube was purged with high-purity Ar gas with a constant flow rate of 300 sccm. Lastly, the tube was slowly cooled down to room temperature. For the growth of hierarchical nanostructures, an 8-nm-thick Au film was directly coated on the as-grown CdS NWs by thermal evaporation. Then, CdTe nanostructures were grown on the CdS NWs using the same method as described above.

### Fabrication Process of Photodetectors Based on Hierarchical Nanostructures

The as-grown CdTe NWs or CdTe/CdS hierarchical nanostructures were dispersed in anhydrous ethanol by ultra-sonication for ~2 min. And then, using a drop and evaporation process, the individual NWs or the hierarchical nanostructures were adsorbed on a clean bare SiO_2_ substrate. Lastly, metal electrodes (Au/Ti = 50 nm/10 nm) were fabricated by a conventional lift-off process.

## Results and Discussion

**Fig. 1:**
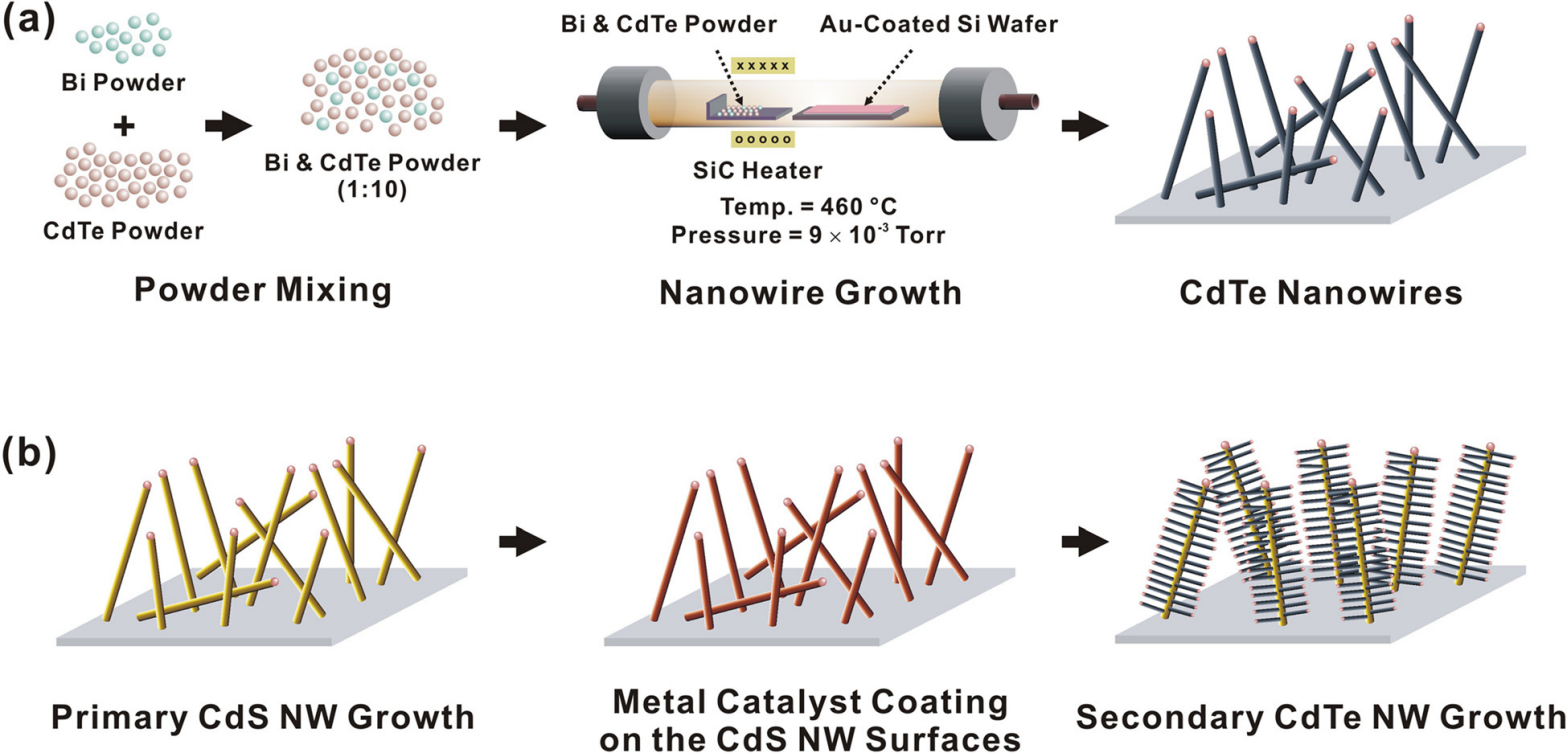
Schematic diagrams depicting the growth process of CdTe nanostructures. **a** Bi-assisted CdTe nanostructure growth by a CVD method. **b** Growth of CdTe/CdS hierarchical nanostructures

Figure 1a shows the schematic diagram depicting the method to grow CdTe NWs. The NWs were grown in a horizontal tube furnace via a vapor-liquid-solid (VLS) process. In brief, CdTe powder was mixed with Bi powder in a 10:1 weight ratio. The mixed powder was placed at the center of an alumina tube furnace as a source material, while an 8-nm-thick Au or Bi film-coated SiO_2_ substrate was placed at the downstream position of the source material. For the growth of the CdTe NWs, the furnace tube was rapidly heated up to a target temperature of 460~540 °C at a rate of 32.5 °C/min under a constant Ar flow. When the temperature reached the target temperature, the furnace tube was evacuated to a base pressure of 8 × 10^−3^ Torr. Then, the furnace tube was kept at a constant temperature and pressure condition for a controlled time (90~120 min). At the end of the growth process, the tube was thoroughly purged with a constant Ar flow and cooled down slowly.

Figure 1b shows a schematic diagram depicting the fabrication of hierarchical nanostructures via secondary growth of CdTe nanostructures on CdS NWs. Firstly, single-crystalline CdS NWs were grown on a catalyst-coated SiO_2_ substrate using a previously reported VLS method [12–14]. The detailed process is described in our previous report [13]. For the growth of hierarchical nanostructures, an 8-nm-thick Au film was directly coated on the as-grown CdS NWs by thermal evaporation. Then, CdTe nanostructures were grown on the CdS NWs using the same method shown in Fig. 1a. Note that since we utilized a high-temperature VLS method to grow the secondary nanostructures, the number of impurities at the interface between CdS NWs and CdTe nanostructures should be minimal, which is advantageous in improving the optoelectronic performance of our hierarchical nanostructures [12, 14].

**Fig. 2:**
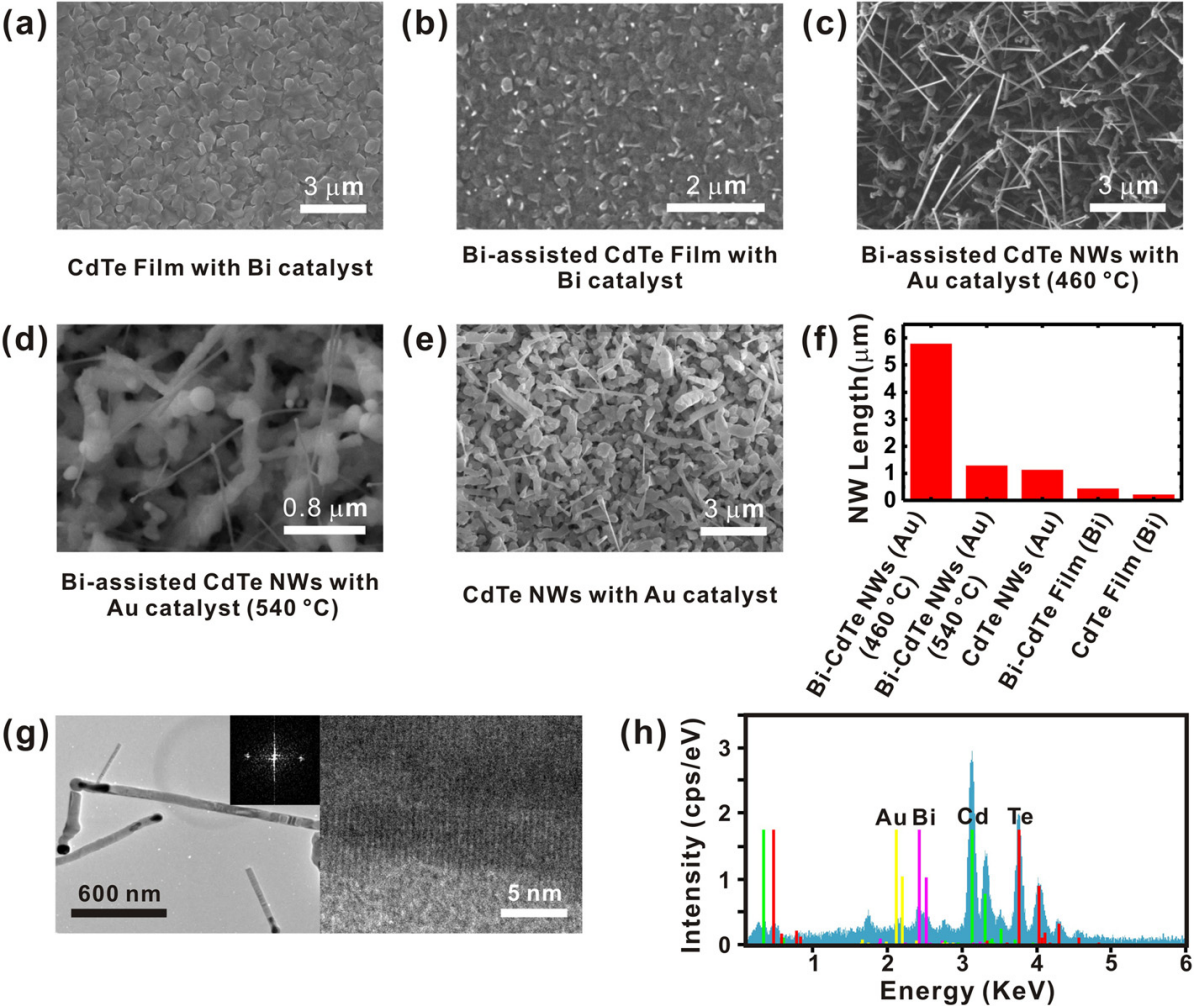
Structural control of CdTe nanostructures. **a** CdTe film grown on a Bi-coated film. **b** Bi-assisted CdTe film grown on a Bi-coated film. **c** Bi-assisted CdTe NWs grown on a Au-coated film at 460 °C. **d** Bi-assisted CdTe NWs grown on a Au-coated film at 540 °C. **e** CdTe NWs grown on a Au-coated film. **f** Average lengths of CdTe NWs grown at different conditions. **g** TEM images of CdTe NWs. The *right-hand image* shows a magnified TEM image measured on a single CdTe NW. The *upper inset* shows the diffraction pattern of the CdTe NW. **h** EDS measurement of as-grown CdTe NWs

Figure 2a–e shows the scanning electron microscopy (SEM) images of various CdTe nanostructures grown under different growth conditions. First, when we used a pure CdTe powder source and Bi film catalyst to grow the nanostructures, we obtained a CdTe thin film on the substrate as shown in Fig. 2a. On the other hand, when we adopted a mixed powder source including both CdTe and Bi (10:1 weight ratio), we obtained CdTe NWs (Fig. 2b).

However, the NWs were quite short, and most of them were just in an initiation phase. Here, the length of the CdTe NWs could be extended by replacing the Bi catalyst with Au film catalyst. Figure 2c shows a SEM image of CdTe NWs grown at 460 °C using the mixed powder (CdTe:Bi = 10:1 weight ratio) as a source material and an 8-nm-thick Au film as a catalyst instead of Bi catalyst. Notably, the CdTe NWs had a much higher aspect ratio than pure CdTe NWs grown on Bi catalysts (Fig. 2b). On the other hand, the aspect ratio of CdTe NWs decreased as the growth temperature was increased up to 540 °C (Fig. 2d). Therefore, we chose 460 °C as the growth temperature for the synthesis of CdTe NWs with a high aspect ratio. As a control experiment, we have also grown CdTe nanostructures using Au catalyst and a pure CdTe powder source (without Bi powder). In this case, just a small number of NWs was grown as shown in Fig. 2e. The length and diameter of the NWs were not uniform, and the aspect ratio was very small. This result implies that Bi catalyst can make a huge difference in the morphology of the nanostructures as well as their structural uniformity during the growth of CdTe nanostructures [27]. Similarly, it has been reported that the Bi powder induces the asymmetric crystallization of CdTe nanowires, which enabled the directional growth of NWs [28].

The average lengths of CdTe NWs grown under the different conditions are shown in Fig. 2f. This clearly shows that we can deliberately control the NW structures using different catalysts and growth conditions. In particular, it should be noted that we obtained the longest NWs when we used the Bi-assisted growth with Au catalyst.

Figure 2g shows the high-resolution transmission electron microscopy (HR-TEM) image of as-grown CdTe NWs (Fig. 2c). The NWs exhibited alloy droplets as nucleation seeds on their heads, which indicates that the NWs were actually grown by the VLS mechanism. The length of the NWs was several micrometers, and its diameter was about 60 nm. The TEM image shows the ~0.37-nm lattice fringe corresponding to the atomic planes along the [111] lattice direction. This result shows that our process allows us to grow highly crystalline CdTe NWs. The X-ray diffraction analysis suggests that the NWs had a cubic structure with the lattice parameter of *a* = 6.410 Å (Figure S1 in Additional file 1). The energy-dispersive X-ray spectroscopy (EDS) result of as-grown CdTe NWs (Fig. 2h) shows that the CdTe NWs were chemically pure, and the stoichiometric ratio was Bi:Cd:Te = 1:12:11.

**Fig. 3:**
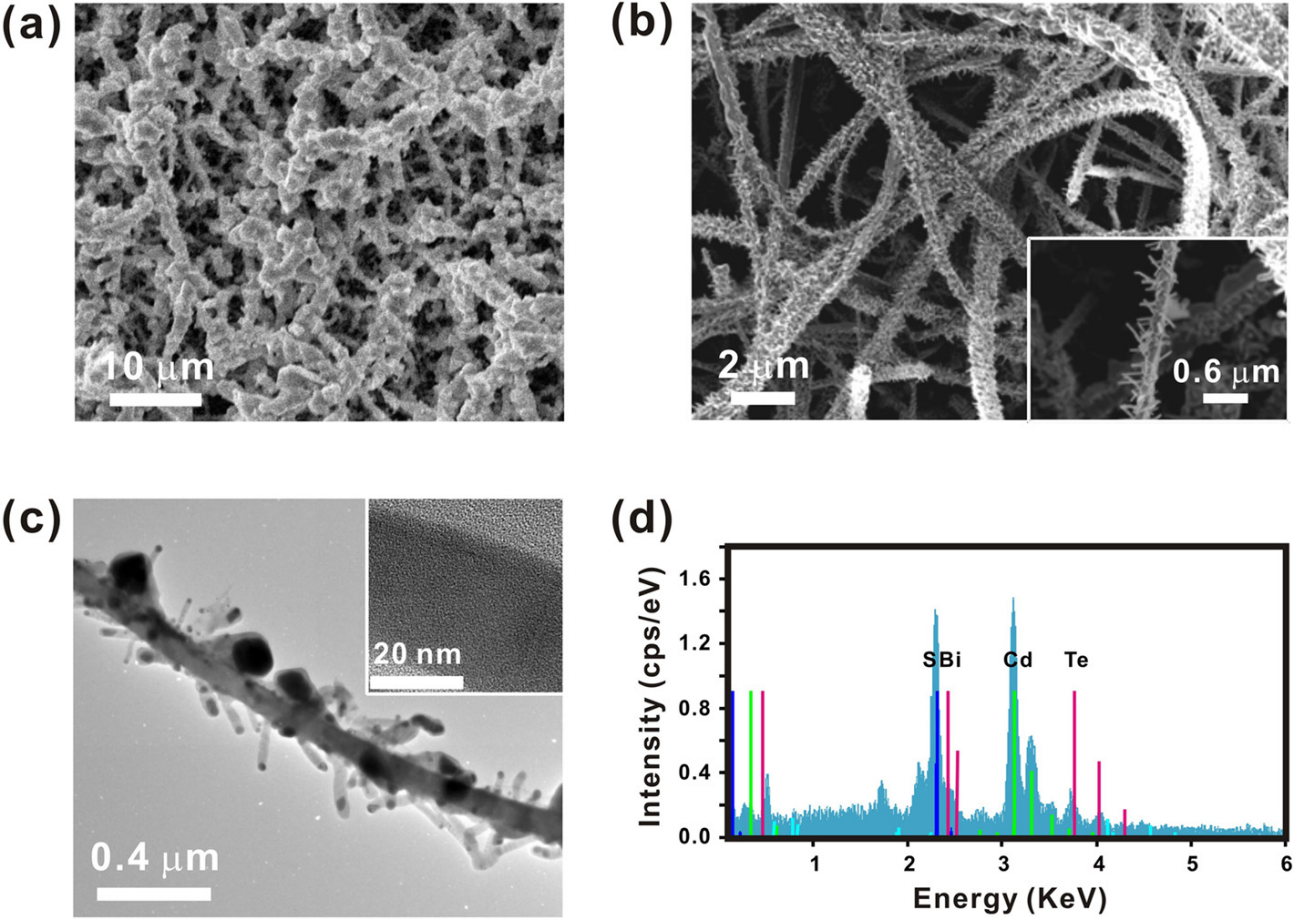
Fabrication of CdTe/CdS hierarchical nanostructures. **a** SEM images of CdTe-coated CdS NWs. **b** SEM images of CdTe/CdS hierarchical nanostructures. **c** TEM images of CdTe/CdS hierarchical nanostructures. The *inset* shows the magnified TEM image of CdTe NWs. **d** EDS measurement of CdTe/CdS hierarchical nanostructures

One of the advantages of the Bi-assisted CdTe nanostructures is that they can be directly utilized as building blocks for various hierarchical nanostructures. We demonstrated the fabrication of hierarchical nanostructures consisting of n-type CdS NWs and p-type CdTe NWs (or films). The detailed fabrication process is described in the “Methods” section. First, when pure CdTe powder source and Bi catalyst were used to grow the secondary nanostructures, we could obtain the CdS NWs coated with CdTe films (Fig. 3a). On the other hand, when we used the mixed powder (CdTe:Bi = 10:1) as a source material and an 8-nm-thick Au film as a catalyst, we could obtain a hierarchical nanostructure composed of CdTe NWs branched on the seeding CdS NWs (CdTe/CdS NWs) as shown in Fig. 3b. Note that this result is consistent with that shown in Fig. 2a, c.

A TEM image of a single as-grown branched CdTe/CdS NW is shown in Fig. 3c. The branched CdTe NWs were randomly oriented on the backbone CdS NW. The lattice fringe of the CdTe NWs was spaced by ~0.36 nm, which indicates the NWs had a high-quality crystalline structure. Figure 3d shows an EDS spectrum taken from our CdTe/CdS hierarchical nanostructures. Through EDS analysis, we could confirm that each of the CdS NWs and CdTe NWs was chemically pure and composed only of cadmium (Cd), sulfur (S), and telluride (Te) materials.

This process can be applied to various nanostructures other than CdTe/CdS NW hierarchical nanostructures. For example, we could also successfully fabricate CdS NW-CdS NW hierarchical structures (Figure S2 in Additional file 1), CdTe thin films, and CdTe NW-CdS nanobelt (NB) hierarchical structures (Figure S3 in Additional file 1).

**Fig. 4:**
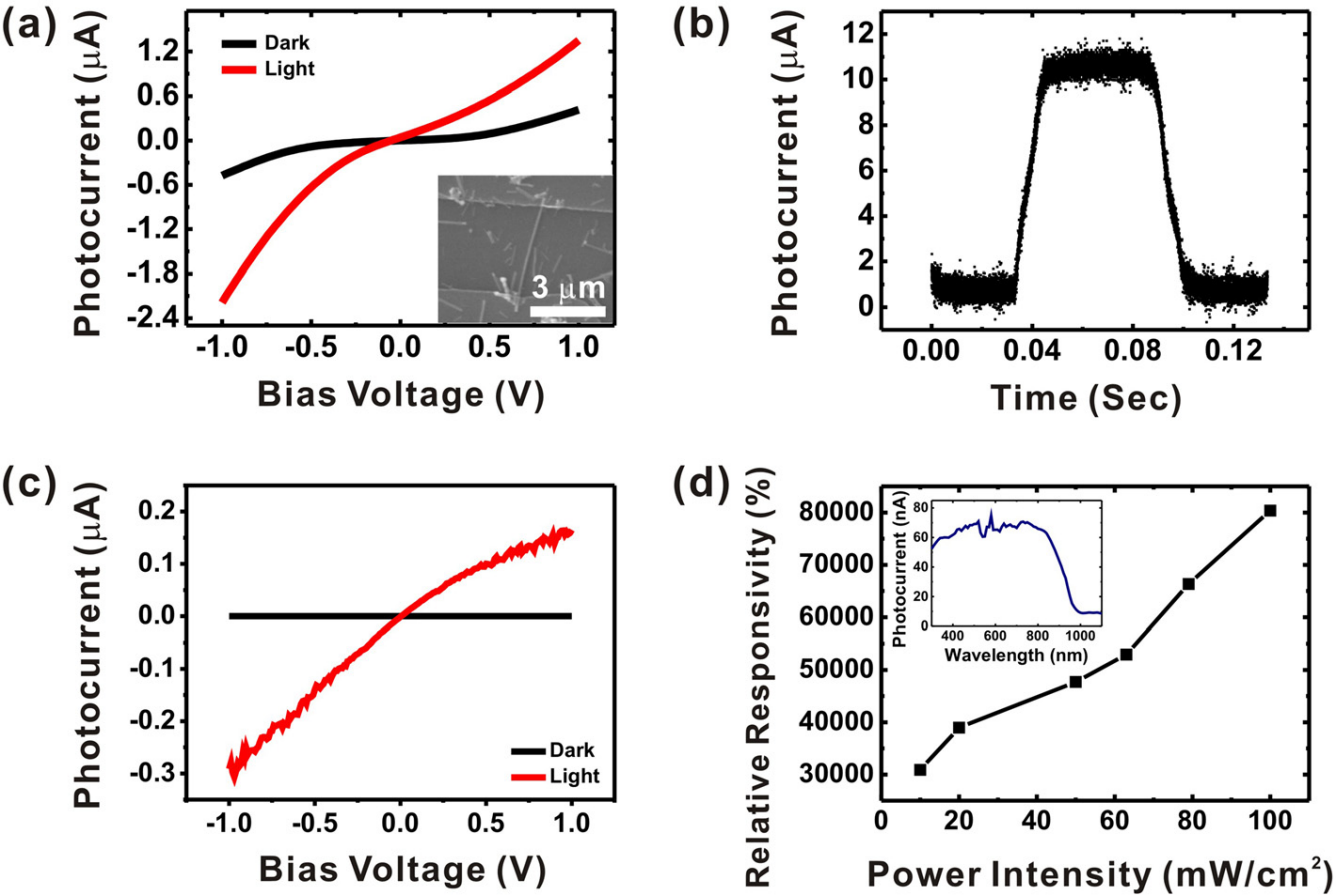
Photoresponse characteristics of devices based on a single CdTe NW or CdTe/CdS hierarchical nanostructures. **a** *I*-*V* characteristics of a photodetector based on a single CdTe NW with (*red*) or without (*black*) white light illumination. **b** Single modulation cycle of the photodetector based on CdTe NW exposed to a white light of 100 mW/cm^2^ at a bias voltage of 1 V. **c** *I*-*V* characteristics of a photodetector based on CdTe/CdS hierarchical nanostructures with (*red*) or without (*black*) a white light illumination. **d** Relative responsivity of a photodetector based on CdTe/CdS hierarchical nanostructures. The *inset* shows the spectral response of the CdTe/CdS hierarchical nanostructure device measured at a bias voltage of 1 V

Figure 4a, b shows the photoresponse characteristics of a photodetector based on a single CdTe NW. The fabrication process is shown in the “Methods” section. The channel length of the NW junction is 4 μm as shown in the inset image of Fig. 4a. The metal electrodes consist of Au and Ti (60 nm/10 nm). Figure 4a shows the *I*-*V* curve of the CdTe NW photodetector with and without light illumination. Here, *I*_light_ and *I*_dark_ represent the currents with and without the light source, respectively. For the photocurrent measurement, we utilized a solar simulator (Newport 91160A) as a light source. This light source had a white light spectrum with a power density of 100 mW/cm^2^ (AM 1.5). Optical power filters were utilized to control the intensity of the light from the solar simulator. The measured photocurrent *I*_p_, defined as *I*_light_ − *I*_dark_, was 1.71 and 0.94 μA at the bias voltage of −1.0 and +1.0 V, respectively. The asymmetrical photocurrent behavior is probably due to defects or impurities which can generate an intrinsic potential difference between two metal electrodes [29].

We also measured the photocurrent with different incident optical power densities. This photocurrent behavior can be fitted by a simple power law [10, 13, 14, 30]:

$$ {I}_{\mathrm{p}}=A\cdot {P}^x $$

where *I*_p_, *A*, and *P* represent the photocurrent, the proportionality constant, and the power of a light source, respectively. The fitting result showed *x* = 0.96. The experimental data is shown in Figure S4 in Additional file 1. The value of the exponent *x* close to 1 indicates the low density of trap states in the energy band gap between the Fermi level and the conduction band edge of NWs [31]. The relative responsivity ((*I*_light_ − *I*_dark_)/*I*_dark_%) can be also obtained from the photocurrent data. The maximum relative responsivity was estimated as ~780 % (Figure S4 in Additional file 1). The detectivity D* of the CdTe NW photodetector can be also calculated by a well-known equation [32, 33]:

$$ {D}^{*}\kern0.5em =\kern0.5em \mathrm{Responsivity}\kern0.5em /\kern0.5em \sqrt{2\kern0.5em e\kern0.5em {I}_{\mathrm{dark}}\kern0.5em /\kern0.5em \mathrm{area}}\kern0.5em =\frac{I_{\mathrm{p}}\kern0.5em /\kern0.5em \left(P\kern0.5em \cdot \kern0.5em \mathrm{area}\right)}{\sqrt{2\kern0.5em e\kern0.5em {I}_{\mathrm{dark}}\kern0.5em /\kern0.5em \mathrm{area}}} $$

where *P* and *e* are the power of the light source and the absolute value of electron charge, respectively. The estimated detectivity is 7.996 × 10^11^ cm Hz^1/2^ W^−1^ and 4.698 × 10^11^ cm Hz^1/2^ W^−1^ at the bias voltage of −1.0 and +1.0 V, respectively.

Figure 4b shows the time response of a CdTe NW photodetector. A xenon lamp (300 W) and a mechanical chopper were used to provide the intensity-modulated input light, while a 1 V bias voltage was applied to the photodetector. Here, we measured the rise and fall times which were defined as the times required for the current to increase from 10 to 90 % of *I*_light_ and decrease from 90 to 10 % of *I*_light_, respectively. In the case of the photodetector based on CdTe NWs, the measured rise time was 9.45 ms, and the fall time was 9.98 ms.

To enhance the relative photoresponsivity, we also fabricated photodetectors using CdTe/CdS hierarchical nanostructures. The fabrication method was similar to that of photodetectors based on a single CdTe NW. Figure 4c shows the *I*-*V* curves of the hierarchical nanostructure-based photodetector with or without light illumination. The measured photocurrent *I*_p_ was 0.29 and 1.65 μA at the bias voltage of −1.0 and +1.0 V, respectively. The relative responsivity of the photodetector based on hierarchical nanostructures exhibited values in the range of 30,000~80,000 % as shown in Fig. 4d. The detectivity was estimated as 8.585 × 10^12^ cm Hz^1/2^ W^−1^ and 4.039 × 10^13^ cm Hz^1/2^ W^−1^ at the bias voltage of −1.0 V and +1.0 V, respectively. Additionally, the photoconductive responses of the photodetectors based on the hierarchical nanostructures were also measured with different incident optical powers (Figure S5 in Additional file 1). It should be noted that all of these photoresponse performances such as the photocurrent, the detectivity, and the relative responsivity were quite improved compared to those of photodetectors based only on a single CdTe NW. Presumably, this extreme enhancement of the responsivity was due to the PNP type junction formed at CdTe NW (p-type)/CdS NW (n-type)/CdTe NW (p-type) interfaces. The PNP junction could decrease *I*_dark_ more effectively, resulting in enhanced relative responsivity.

Additional file 1: Figure S6 shows the time response of the hierarchical nanostructure-based photodetector. This time response was measured using the same method as that used for the photodetector based on single CdTe NWs. When repeated light pulses with a frequency of 10 Hz were applied, the photodetector exhibited stable photoresponse without any degradation. Although this NW-based photodetectors did not show an ultra-fast responsivity, the stable response to a pulsed light source showed a possibility of using the CdTe/CdS hierarchical nanostructure as a component for practical photodetector applications.

The inset in Fig. 4d depicts the spectral response of the hierarchical nanostructure-based photodetector. For the measurement, a xenon lamp (100 mW cm^−2^) and a monochromator (Acton Research Corporation SpectraPro-150) were used to provide the monochromatic light with a wavelength ranging from 300 to 1100 nm. The photocurrent was measured at a 1 V bias voltage. The photocurrent was quite small for the light with its wavelength longer than 950 nm. When the wavelength approached values shorter than 950 nm, the photocurrent increased slowly and had a maximum value near 800 nm. This edge corresponds to the band-edge emission of CdTe NWs (*Eg* ≈ 1.5 eV, 827 nm, 300 K) [34], revealing that the photoconductivity was due to the electron-hole pairs generated by the incident light with a larger energy than the band gap. Unlike the spectral response of single CdTe NW-based devices [35], the increased photocurrent showed a plateau up to 400 nm and decreased beyond wavelength values shorter than 400 nm. Presumably, the absorption over a broad wavelength range in our devices was due to the heterojunction composed of CdTe and CdS NWs.

## Conclusions

We developed a simple method to control the structure of CdTe NWs using Bi powder in a standard CVD process, which enabled the fabrication of NW-based hierarchical nanostructures with a largely enhanced photoconductivity. As a proof of concepts, we fabricated a photodetector based on CdTe/CdS hierarchical nanostructures. The hierarchical nanostructure-based photodetector showed a ~100 times higher photoresponsivity than photodetectors based only on a single CdTe NW. This improved photoconductivity is presumably due to the intrinsic PNP junction electronic structure of our branched NW-based hierarchical nanostructures. Our simple but efficient strategy should enable the fabrication of high-performance optoelectronic devices for versatile practical applications.

## Additional file

Additional file 1:
**Supplementary figures (Figures S1–S6).**(DOCX 763 kb)
