# Supplementary material for: Bi-Assisted CdTe/CdS Hierarchical Nanostructure Growth for Photoconductive Applications
Source: Nanoscale Res Lett. 2015 Aug 19;10:331. doi: 10.1186/s11671-015-1037-6 (PMC4539310; doi:10.1186/s11671-015-1037-6)
Supplement: Additional file 1: — Supplementary figures (Figures S1–S6).(DOCX 763 kb) [file 11671_2015_1037_MOESM1_ESM.docx]

**Additional file 1**

Bi-assisted CdTe-CdS Hierarchical Nanostructure Growth for Photoconductive Applications

Kwang Heo^1,2#^, Hyungwoo Lee^1#^, Jikang Jian^3^, Dong-Jin Lee^4^, Yongju Park^5^, Changhee Lee^5^, Byung Yang Lee^4^* and Seunghun Hong^1,6^*

^1^Department of Physics and Astronomy, Seoul National University, Seoul 151-747, Republic of Korea

^2^Department of Nanotechnology and Advanced Materials Engineering, Sejong University, Seoul 143-747, Republic of Korea

^3^School of Physics and Optoelectronic Engineering, Guangdong University of Technology, Guangzhou 510006, China

^4^School of Mechanical Engineering, Korea University, Seoul 136-713, Republic of Korea

^5^School of Electrical and Computer Engineering, Seoul National University 151-744, Seoul, Republic of Korea

^6^Department of Biophysics and Chemical Biology, Seoul National University, Seoul 151-742, Republic of Korea

E-mail: [blee@korea.ac.kr](mailto:blee@korea.ac.kr), [seunghun@snu.ac.kr](mailto:seunghun@snu.ac.kr)

^#^These authors contributed equally to this work.


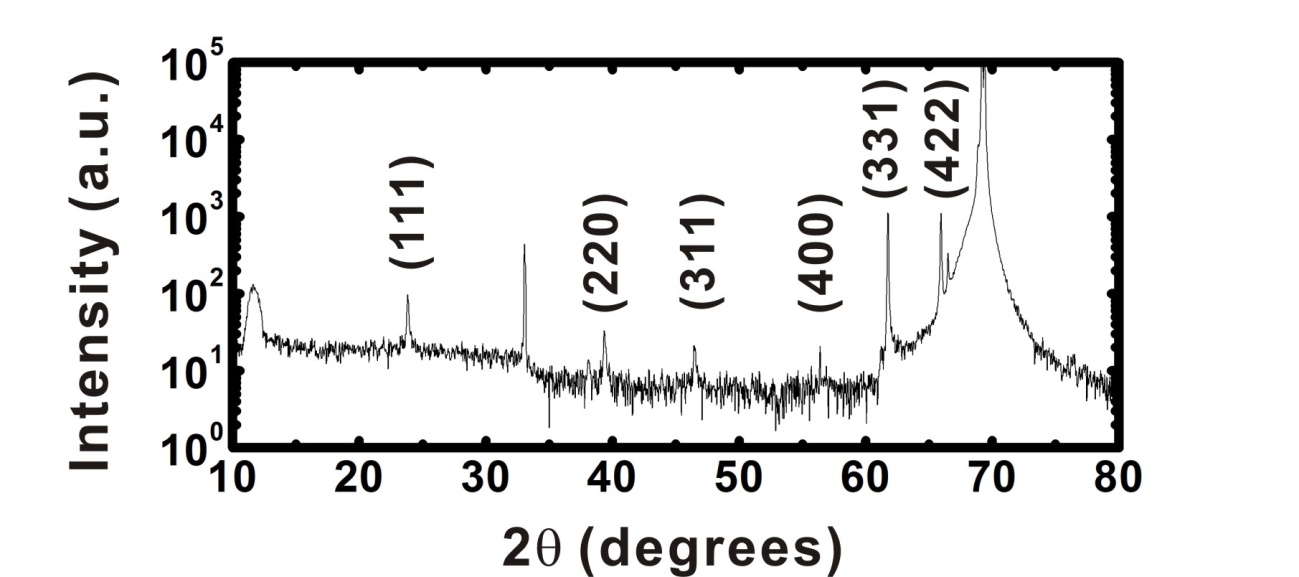


**Figure S1.** XRD pattern of CdTe nanowires (NWs).


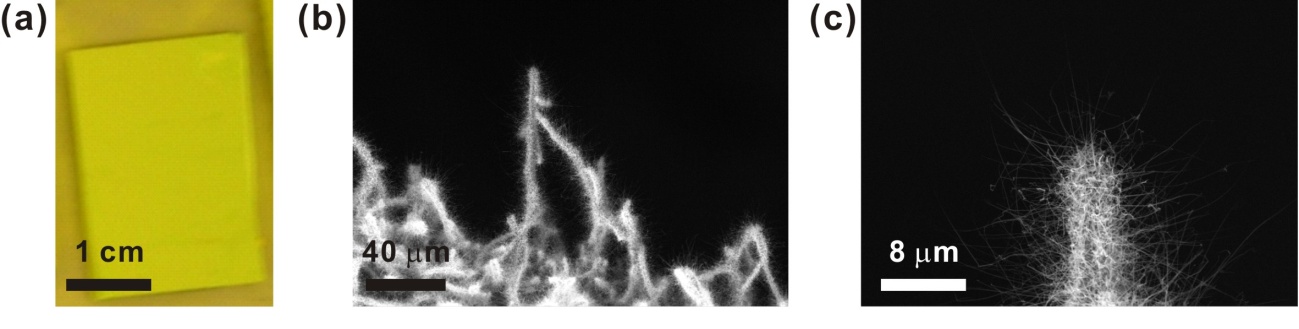


**Figure S2.** (a) Optical image of as-grown CdS NW-CdS NW hierarchical nanostructures. (b) SEM image of the same nanostructures and (c) the magnified image of a single hierarchical nanostructure.


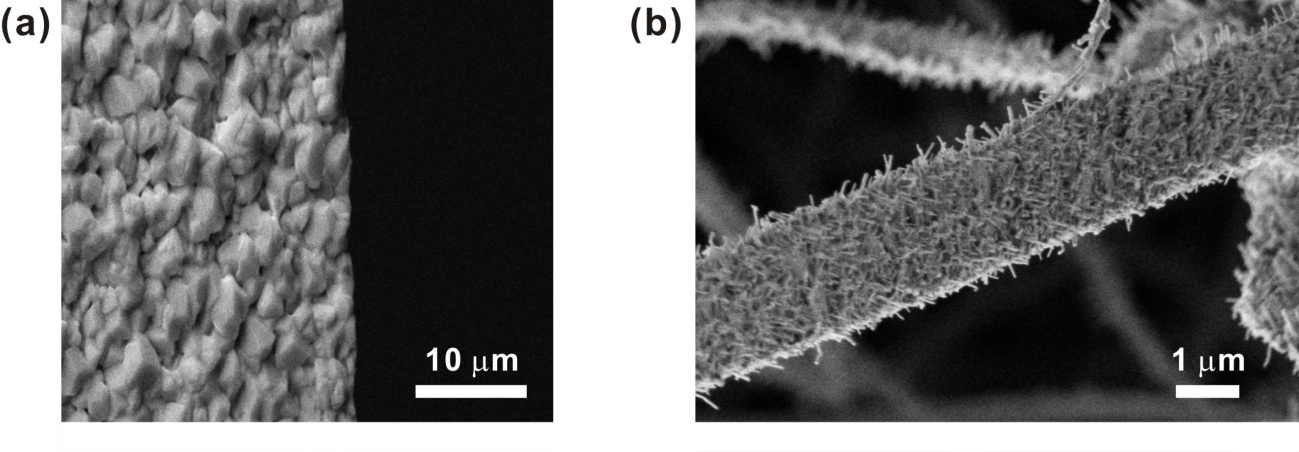


**Figure S3.** (a) SEM image of a CdTe thin film. (b) SEM image of CdTe NW-CdS nanobelt (NB) hierarchical nanostructures. Notably, NWs from the secondary growth had much smaller dimensions than backbone CdS NBs.


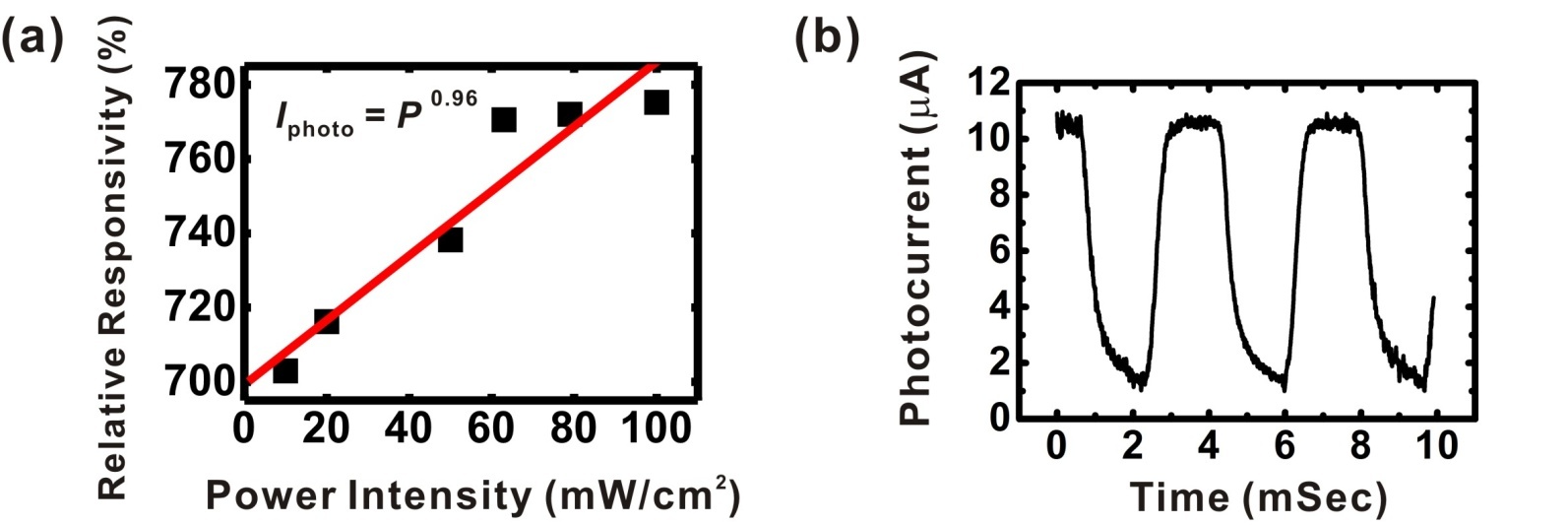


**Figure S4.** Photoconductive characteristics of photosensor devices based on a single CdTe NW. Relative photoresponsivity as a function of the optical power density at a 1 V bias voltage.

**
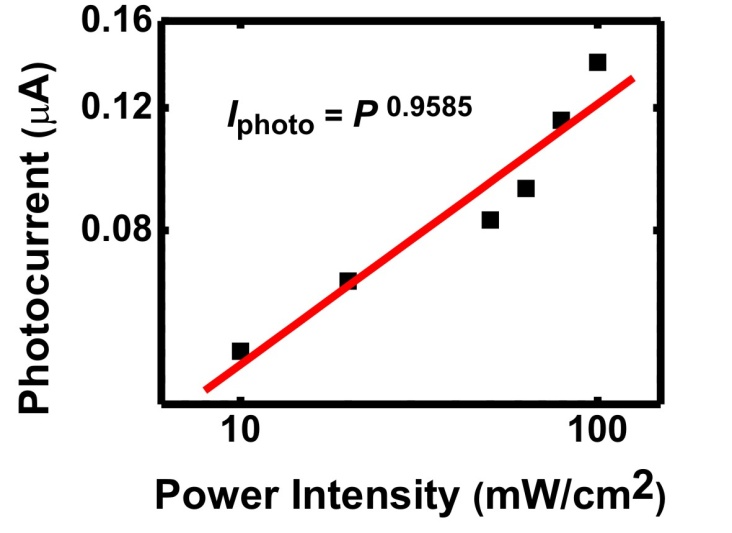
**

**Figure S5.** Photoconductive characteristics of the CdTe/CdS hierarchical nanostructure-based device. Relative photoresponsivity as a function of the optical power density at a +1 V bias voltage.


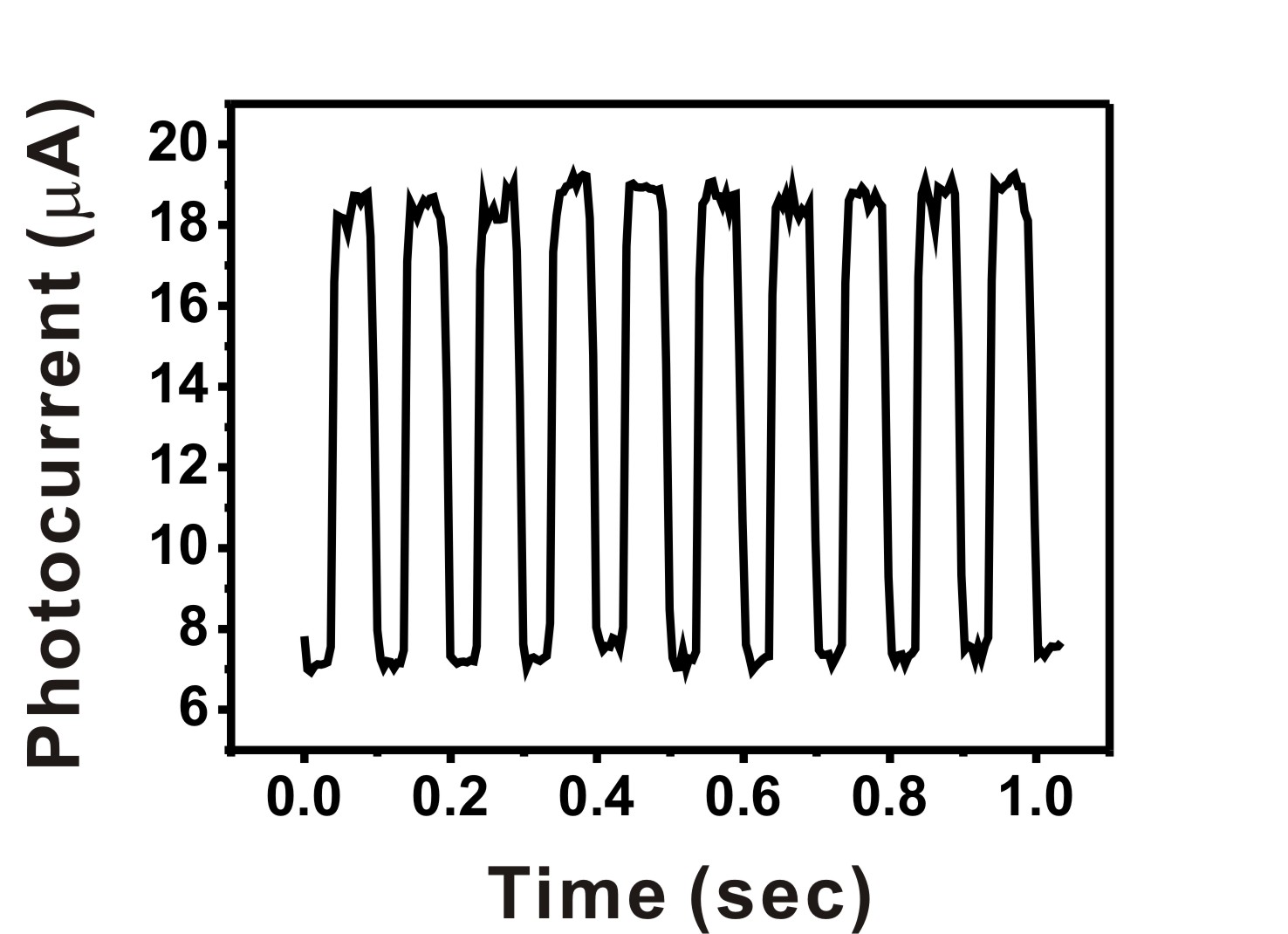


**Figure S6.** Photoresponse and recovery of the CdTe/CdS hierarchical nanostructure device exposed to a white light of 100 mW/cm^2^ at a bias of 1 V.
